# Local structural modelling and local pair distribution function analysis for Zr–Pt metallic glass

**DOI:** 10.1038/s41598-024-64380-2

**Published:** 2024-06-10

**Authors:** Akihiko Hirata, Satoru Tokuda, Chihiro Nakajima, Siyuan Zha

**Affiliations:** 1 Department of Materials Science, Waseda University, Shinjuku, Tokyo 169-8555 Japan; 2 Kagami Memorial Research Institute for Materials Science and Technology, Waseda University, Shinjuku, Tokyo 169-0051 Japan; 3 WPI Advanced Institute for Materials Research, Tohoku University, Sendai, Miyagi 980-8577 Japan; 4 Mathematics for Advanced Materials-OIL, AIST, Sendai, Miyagi 980-8577 Japan; 5 Center for Basic Research on Materials, National Institute for Materials Science, Tsukuba, 305-0047 Japan; 6 Research Institute for Information Technology, Kyushu University, Kasuga, Fukuoka 816-8580 Japan; 7 Institute of Mathematics for Industry, Kyushu University, Fukuoka, 816-8580 Japan; 8 Faculty of Science and Technology, Tohoku Bunka Gakuen University, Sendai, 980-8551 Japan

**Keywords:** Characterization and analytical techniques, Glasses

## Abstract

In disordered glass structures, the structural modelling and analyses based on local experimental data are not yet established. Here we investigate the icosahedral short-range order (SRO) in a Zr–Pt metallic glass using local structural modelling, which is a reverse Monte Carlo simulation dedicated to two-dimensional angstrom-beam electron diffraction (ABED) patterns, and local pair distribution function (PDF) analysis. The local structural modelling invariably leads to the icosahedral SRO atomic configurations that are similarly distorted by starting from some different initial configurations. Furthermore, the SRO configurations with 11–13 coordination numbers reproduce almost identical ABED patterns, indicating that these SRO structures are similar to each other. Further local PDF analysis explicitly indicates the presence of the wide distribution of atomic bond distances, which is comparable to the global PDF profile, even at the SRO level. The SRO models based on the conventional MD simulation can be strengthened by comparison with those obtained by the present local structural modelling and local PDF analysis based on the ABED data.

## Introduction

Metallic glasses have been widely developed and applied to structural and functional materials due to their various good properties such as strength, corrosion resistance, and soft magnetism^1^. Understanding the short-range order (SRO) is essential to reveal atomistic mechanism of the superior properties, because the SRO structures are the fundamental structural units that generate the entire glass structures. In addition, some properties may arise from the bonding nature associated with the SRO structures. For example, it has been suggested that the orbital hybridization, which induces bond shortening in metallic glasses, is a key factor in determining the mechanical properties^2^. Moreover, the bonding nature should be influenced by the coordination numbers, which are basically governed by the atomic size ratio and have a broad distribution^3–11^. These structural details are essentially buried in spatially averaged global experimental data such as a pair distribution function (PDF). In other words, broad peaks in experimentally obtained global PDFs for metallic glasses could be related to both the bond shortening (or stretching) and the broad coordination number distribution. However, there is still no clear answer as to how such details of individual SRO structures in metallic glasses correlate with the features of global PDFs. To solve this problem, the development of analytical tools for sub-nanometer scale SRO structures is highly needed.

Recently, we have attempted to detect SRO in glasses using scanning transmission electron microscopy, based on the sub-nanometer scale angstrom-beam electron diffraction (ABED) method^12^. Using this technique, we observed two-dimensional diffraction patterns with a discrete intensity, instead of the halo rings typically observed from a wide area, by reducing the size of the electron beam to about 0.4–0.8 nm. We also used molecular dynamics (MD) simulations to show that these discrete patterns originate from non-crystalline SRO structures in glasses. The local structures for the metallic^12,13^, oxide^14^, and chalcogenide^15^ glasses have been directly observed and analysed using the ABED method. However, the structural evaluation for SRO has not yet been established.

**Figure 1:**
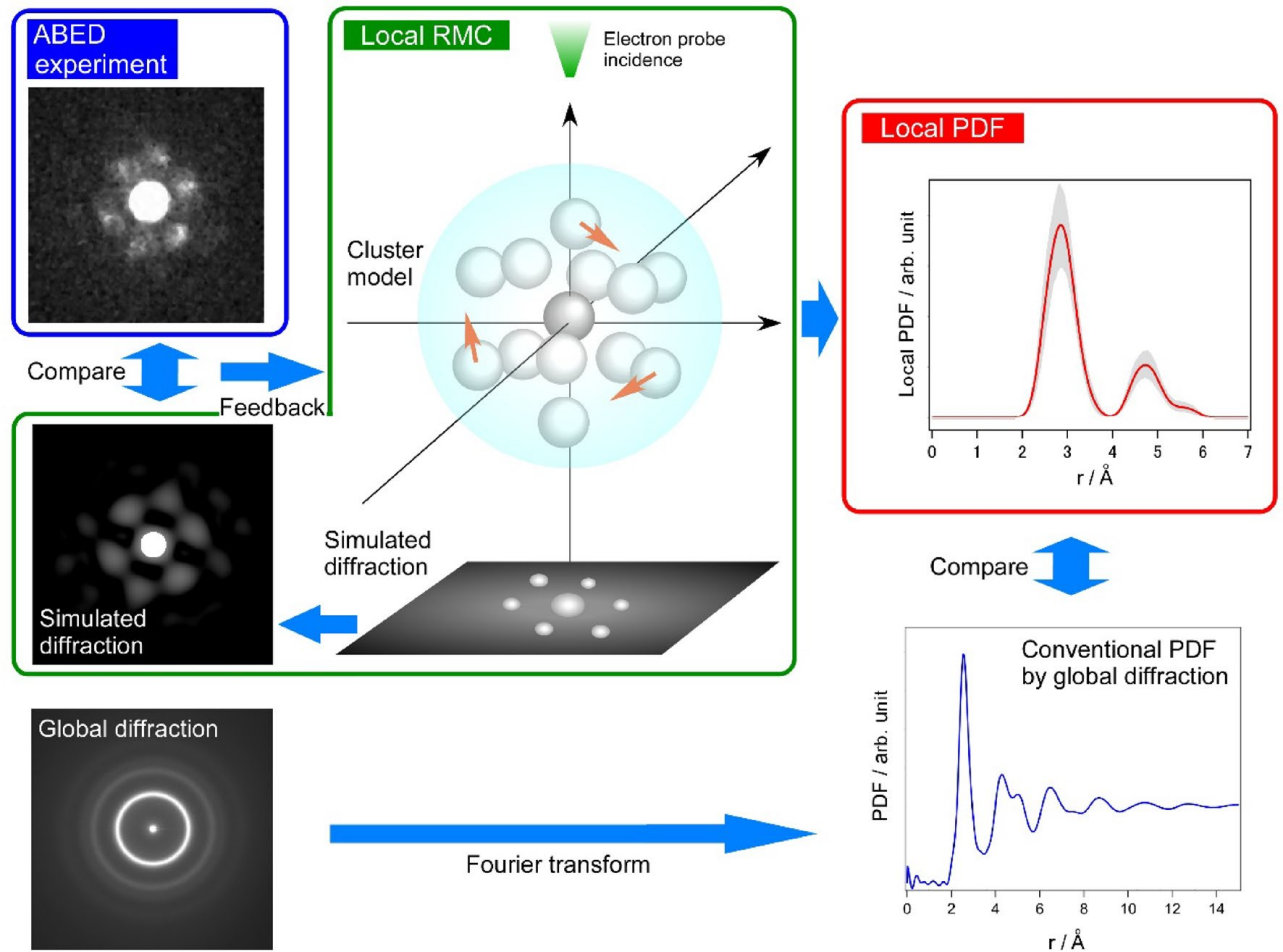
Procedures of a structural analysis for local atomic structures in metallic glasses. The procedures are composed of three parts: 1. angstrom-beam electron diffraction (ABED) experiment, 2. local reverse Monte Carlo (RMC) modelling, and 3. local pair distribution function (PDF) analysis. These three steps are necessary for making experimental-based local PDFs. The local PDFs are compared with the conventionally used global PDFs obtainable from global diffraction data that contain three-dimensionally isotropic intensity distribution.

The PDF analysis has been widely used for the structure analysis of glassy disordered materials. The procedures of the PDF analysis have been established and applied to intensity analyses for X-ray, neutron, and electron diffraction experiments^3^. Using a difference vector ${\varvec{r}}_{mn} \left( { = {\varvec{r}}_{m} - {\varvec{r}}_{n} } \right)$ for the *m*th and *n*th atomic positions in the disordered structure, the three-dimensional total intensity for monatomic systems containing *N* atoms can be written as

1 $$I\left( {\varvec{Q}} \right) = Nf^{2} \left( {\varvec{Q}} \right)\left( {1 + \mathop \sum \limits_{{\begin{array}{*{20}c} {n = 1} \\ {\left( {n \ne m} \right)} \\ \end{array} }}^{N} exp\left( {i{\varvec{Q}} \cdot {\varvec{r}}_{mn} } \right)} \right),$$

where ${\varvec{Q}}$ is a diffraction vector and $f\left( {\varvec{Q}} \right)$ is an atomic scattering factor. Because the intensity obtainable from conventional diffraction experiments are basically isotropic (*see* “global diffraction” in Fig. 1) in a three-dimensional space, the intensity naturally becomes one dimensional information

2 $$I\left( Q \right) = Nf^{2} \left( Q \right)\left( {1 + \mathop \smallint \limits_{0}^{\infty } 4\pi r^{2} \left( {\rho \left( r \right) - \rho_{0} } \right)\frac{sinQr}{{Qr}}dr} \right),$$

where $\rho \left( r \right)$ and $\rho_{0}$ are a density function and an average atomic density, respectively. Note that the scattering vector ${\varvec{Q}}$ in the Eq. (1) is replaced by a scalar $Q$. In addition, for the purposes of clarity and emphasis on the macroscopic versus microscopic distinctions in the intensity analysis, Eqs. (1) and (2) are presented in a simplified form that assumes a single element system. By subtracting a background from the total intensity, it is possible to obtain a PDF profile using Fourier transform. Equation (2) can only be applied to global diffraction including structural information from a large number of atoms, because the intensity should be isotropic. However, for local diffraction intensity from a small number of atoms such as ABED, it is necessary to use Eq. (1) to understand the spotty directional intensity as seen in ABED data (see ABED experiment in Fig. 1). However, there is still no suitable method to obtain reasonable PDF profiles from the local diffraction intensity.

In this work, we apply the local structural modelling similar to a reverse Monte Carlo (RMC) simulation^15^ (see Fig. 1) to the SRO (atomic coordination polyhedra) of metallic glasses based on the ABED experiment and also propose a local PDF analysis for the resultant local structure models. This local structural modelling for the ABED data is called local RMC modelling. Since it is difficult to construct PDFs for “anisotropic” SROs including only a dozen atoms as mentioned above, we use a kernel density estimation to solve the problem. The local PDFs for the resultant local structural models are compared with the conventionally used global PDF, which can be derived from the global diffraction data by Fourier transform. Based on the results, we discuss the structural features of icosahedral-like atomic configurations with different coordination numbers in the glass and the origin of the broadening of peaks in the PDFs. It should be noted that icosahedral atomic configurations in glasses or liquids have been extensively discussed by theoretical and experimental approaches^16–21^.

## Results

**Figure 2:**
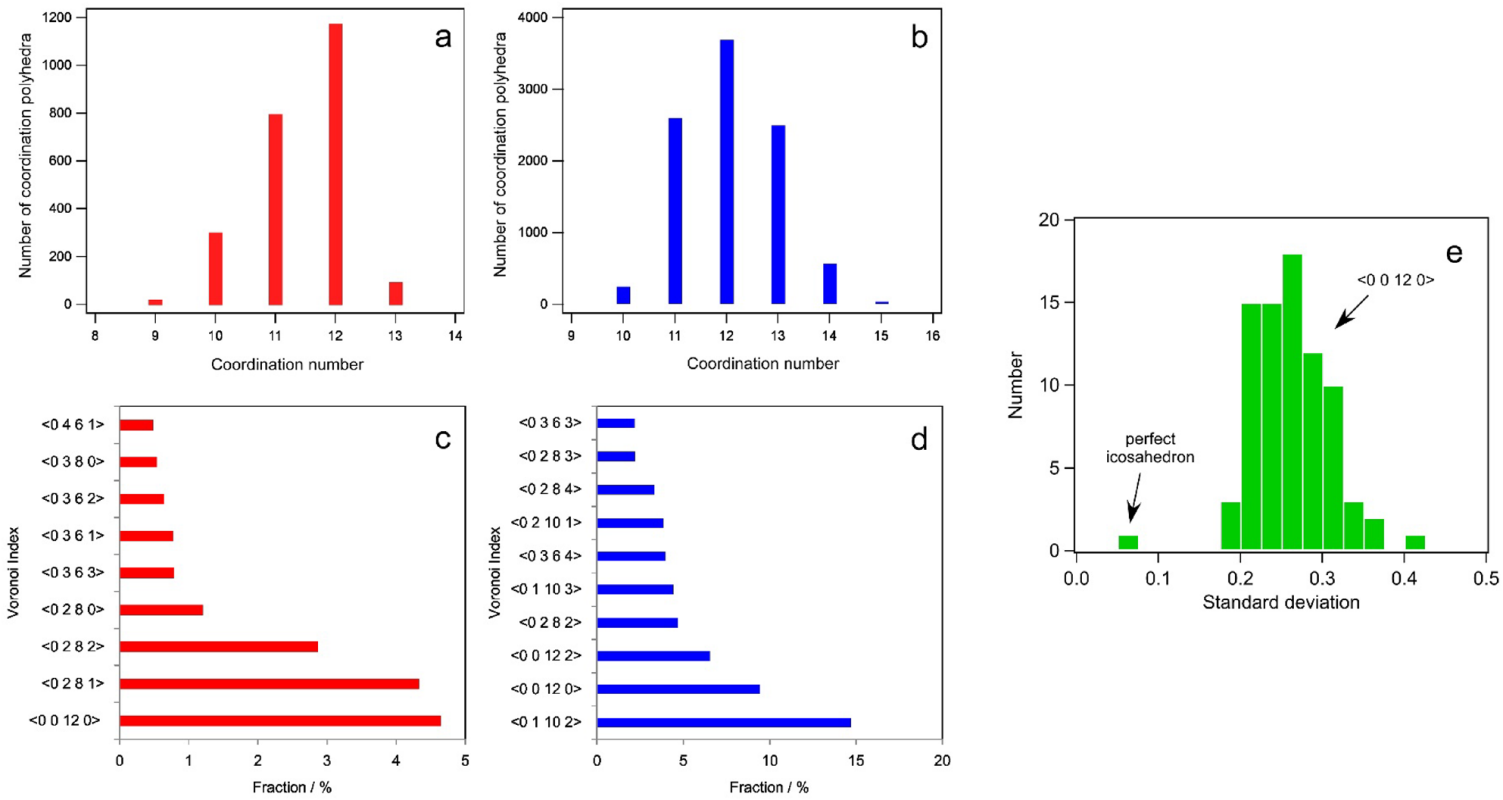
Local atomic environments for molecular dynamics simulation models. Distributions for coordination numbers around (**a**) Pt atoms and (**b**) Zr atoms, and lists of Voronoi indices around (**c**) Pt atoms and (**d**) Zr atoms. Coordination number and Voronoi polyhedral analyses were performed for the classical MD model including 9600 Zr and 2400 Pt atoms. (**e**) Distribution of standard deviation of atomic bond lengths in icosahedral atomic clusters obtained by a MD simulation. A total of 60 icosahedral atomic clusters with Voronoi index of <0 0 12 0> were randomly extracted from the Zr_80_Pt_20_ glass model.

Structural models of metallic glasses for global diffraction data have been confirmed by the complementary use of MD and RMC in the previous studies^3^. We first confirmed a degree of distortion for icosahedral atomic configurations in an MD model. Distributions of coordination numbers and Voronoi polyhedral analyses for the Zr_80_Pt_20_ MD model are shown in Fig. 2. As shown in Fig. 2a,b, the coordination numbers for Pt and Zr are distributed from 9 to 13 and from 10 to 15, respectively. The dominant Voronoi polyhedra are <0 0 12 0> and <0 2 8 1> for central Pt atoms, and <0 1 10 2> and <0 0 12 0> for central Zr atoms, as shown in Fig. 2c,d. Note that the Voronoi index of <0 0 12 0> indicates the presence of an icosahedral atomic configuration with coordination number 12. The standard deviation of atomic bond distances for a MD simulation model is also shown in Fig. 2e. A total of 60 icosahedral atomic clusters with the Voronoi index of <0 0 12 0> were randomly extracted from the Zr_80_Pt_20_ model for statistical analysis. It is evident that the typical icosahedra in the MD model were severely distorted, even though their Voronoi indices are <0 0 12 0> . We also investigated the dependence on the cooling rate, but it did not significantly affect the conclusions (see Fig. S1 in the supplementary material). The trend of distortion will be verified by the local RMC modelling and local PDF analyses as shown below.

The procedure of local RMC modelling based on the ABED experiment and local PDF analysis is summarised in Fig. 1. First, ABED patterns are obtained experimentally as two-dimensional diffraction data from sub-nanometer regions of glassy samples. Then, to obtain three-dimensional SRO structural models by local RMC, we set an initial atomic configuration confined to a spherical boundary whose size is comparable to the experimental beam size. The number density of atoms in the sphere is close to the average number density. The atomic displacement for a randomly selected atom is randomly assigned. A two-dimensional diffraction pattern is calculated for a given configuration by the atomic displacement and then compared with the experimental result by evaluating a judging function. This procedure is repeated until the model matches the experiment, as is the case in a conventional RMC simulation^22–24^. Finally we perform the local PDF analysis for the local structural models obtained by local RMC and compare them with the conventionally used global PDF, which is derived from global “isotropic” diffraction data (halo rings) using Fourier transform. The three-step procedure allows us to obtain reasonable and smooth PDF profiles from local atomic configurations containing as few as a dozen atoms.

**Figure 3:**
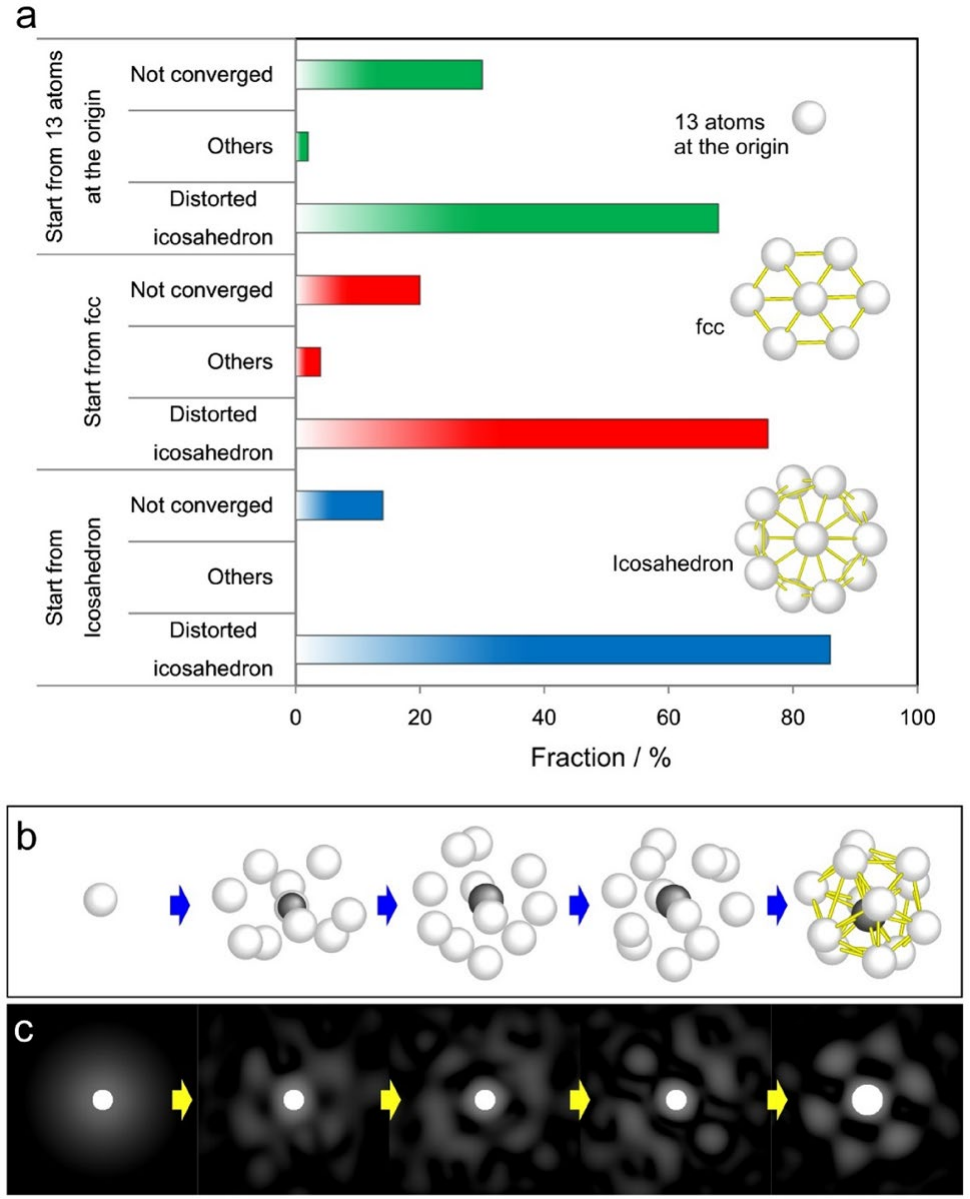
Initial atomic configuration dependency on local RMC modelling. (**a**) Three different initial atomic configurations are prepared: 13 atoms placed at an origin, face-centered cubic (fcc) cluster with 13 atoms, and perfect icosahedral cluster with 13 atoms. The local RMC modeling starts with these three initial configurations and the resultant configurations are listed for the three cases in the graph. The number of trials is 50 for each modeling. “Not converged” means that the calculation got stuck and failed to fit the simulated pattern to the experimental one. (**b**) Formation process of the atomic-structure model satisfying the experiment. (**c**) Simulated ABED patterns calculated from the corresponding structural models of (**b**).

In general, the resultant structural models for the atomistic simulations strongly depend on the initial atomic configurations. Accordingly, we constructed local structural models that are consistent with the experimental diffraction pattern based on three different initial configurations. Three structures, each consisting of 13 atoms with crystalline face-centred cubic (fcc), perfect icosahedron, and structureless configurations were prepared for use in the local RMC modelling. A list of the fractions of final structures constructed by local RMC from the three different initial configurations is shown in Fig. 3a. In all cases, distorted icosahedra with a Voronoi index of <0 0 12 0> were formed, although their initial configurations were completely different from each other. An example of the fitting process for a Zr_80_Pt_20_ metallic glass is shown in Fig. 3b,c. In this case, 13 atoms were placed at the origin as an initial configuration. A diffraction pattern for the initial structure exhibits no diffraction spots due to the structureless feature. The simulated pattern gradually approaches and eventually overlaps with the experimental pattern. The configuration eventually becomes a distorted icosahedron.

**Figure 4:**
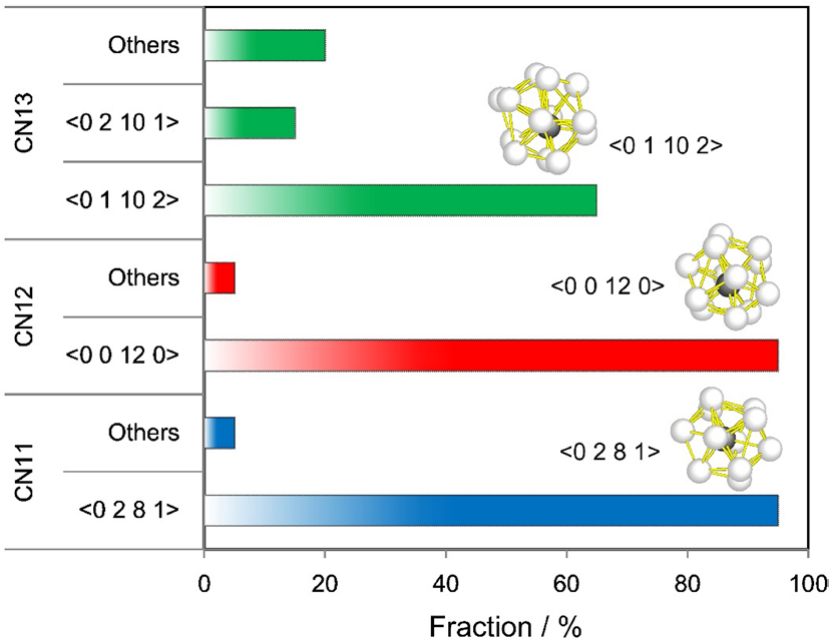
Local reverse Monte Carlo modelling for atomic clusters with different coordination numbers. The final atomic structures after the local RMC modelling started with models consisting of 12, 13, and 14 atoms, corresponding to CN11, CN12, and CN13 atomic clusters, respectively. The number of trials was 20 for each case.

In the above local RMC modelling, the coordination number was fixed at 12. The coordination number, however, is usually not fixed, especially for metallic glass structures with relatively large average coordination numbers, as has been mentioned above. The wide distribution of the coordination numbers for a Zr_80_Pt_20_ MD model obtained by an MD simulation have been already shown in Fig. 2e. We further investigated the effect of the change in coordination number for the fitting results of the local RMC modelling. Figure 4 shows a list of the final structures obtained using the three initial configurations with atomic numbers of 12, 13, and 14 corresponding to CN11, CN12, and CN13, respectively (CN denotes coordination number). All atoms in the initial configurations are placed at the origin. For CN11 and CN12, most attempts resulted in the formation of atomic clusters of <0 2 8 1> and <0 0 12 0> , respectively. For CN13, atomic clusters of <0 1 10 2> and <0 2 10 1> were formed with a total probability of 80%. These results imply that the clusters with <0 2 8 1> , <0 0 12 0> , and <0 1 10 2> (or <0 2 10 1>) adequately match the identical experimental diffraction pattern, indicating a close structural relationship among these atomic clusters. It should also be mentioned that these atomic clusters are frequently found in the MD model, as shown in Fig. 2.

**Figure 5:**
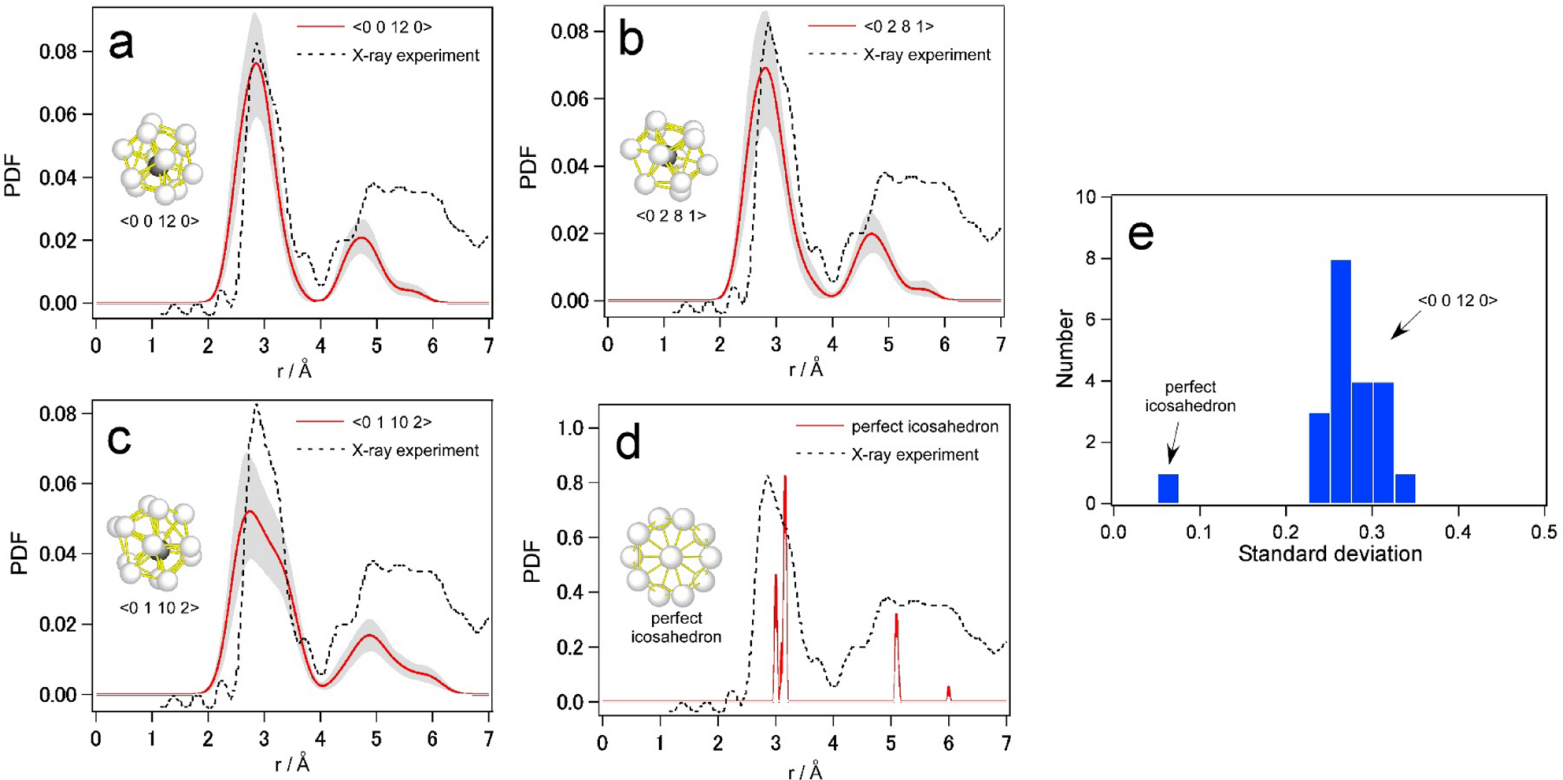
Local pair distribution function analysis. Local PDF profiles for structure models with Voronoi indices of (**a**) <0 0 12 0> (CN12), (**b**) <0 2 8 1> (CN11), (**c**) <0 1 10 2> (CN13), and the perfect icosahedron (CN12). The profile drawn by a dotted line shows the global PDF obtained by X-ray diffraction reported in the literature^25^. (**e**) Distribution of standard deviation of atomic bond lengths in icosahedral atomic clusters.

The local pair distribution function (PDF) profiles for the structural models obtained by local RMC were calculated using a kernel density estimation method. This method allows us to draw reasonable PDF profiles even for atomic clusters consisting of a small number of atoms. Figure 5a shows the PDFs obtained from the final structural model starting from a structureless configuration with 13 atoms, as shown in Fig. 2b. It should be noted that the final structure is a distorted icosahedron with a Voronoi index of <0 0 12 0> . To compare the local information with the global information, a global PDF profile obtained from a conventional X-ray diffraction experiment^25^ is also shown with a dotted line. The distributions of our local models are consistent with those of the X-ray diffraction. This means that each local icosahedron has a similar feature in the bond distance distribution corresponding to the global structure.

The local PDF profiles for the cases of CN11 and CN13 discussed in Fig. 4 are also shown in Fig. 5b,c, respectively. The distributions of bond lengths for CN11 and CN13 are relatively broad, similar to that for CN12 (Fig. 5a). For reference, the local PDF for the perfect icosahedron without any distortion is shown in Fig. 5d. We can immediately see that the width of the peaks in the local PDFs for the metallic glass models are completely different from that of the perfect icosahedron. This implies that the breadth of the global PDF originates from the broad local PDFs of individual SROs, rather than from the broad distribution of coordination numbers, although the distribution for the larger coordination number is slightly broader than that for the smaller coordination number. It can be also interpreted from these facts that the distorted icosahedron with CN12 is completely different from the perfect icosahedron and therefore is only an intermediate between the CN11 and CN13 structures and not a special configuration.

Although most of the final structures have a Voronoi index of <0 0 12 0> for CN12, they are highly distorted and show a wide distribution of atomic bond distances. We then measured the standard deviation of the bond distances for 20 final structural models obtained independently. As shown in Fig. 5e, the standard deviations for all the models are found to be 0.28 ± 0.05 Å, which is much larger than that of a perfect icosahedron. This result implies that the modelling process is highly reproducible, although the atomic displacements are randomly generated in each process. In addition to this, to estimate the atomic displacements between a perfect and distorted icosahedron, we conducted a local RMC modelling started from a perfect icosahedron with small atomic displacements less than 0.1 Å for each step as shown in Fig. S2 in the supplementary material. Therefore, the icosahedral topology was maintained during the modelling process, unlike the cases in Fig. 3. The atomic coordinates before and after the local RMC modelling and the atomic displacements are also given in Table S1 in the supplementary material. The relatively large values of the atomic displacements imply that the icosahedron satisfying an experimental ABED pattern is heavily distorted from the perfect one.

## Discussion

**Figure 6:**
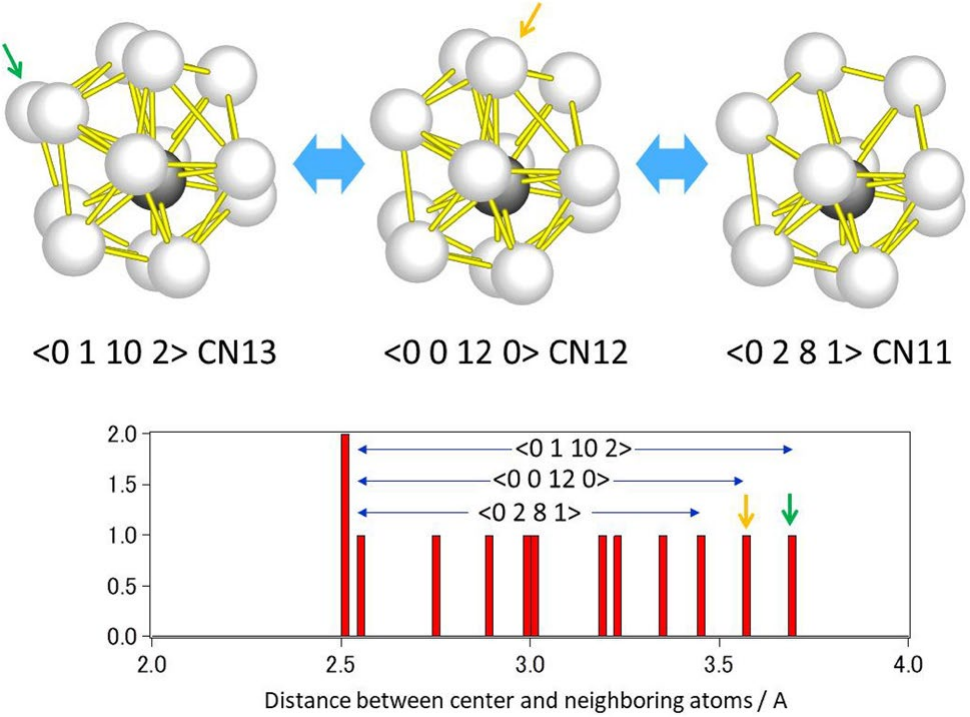
Transition between < 0 1 10 2> and <0 2 8 1> coordination polyhedra via <0 0 12 0> . The polyhedron with <0 1 10 2> (CN13) is transformed into that with <0 0 12 0> by removing the outermost atom (indicated by a green arrow) and further into that with <0 2 8 1> by removing the second-outermost atom (indicated by an orange arrow). Atomic distances between the center and neighboring atoms are also listed in the graph.

**Figure 7:**
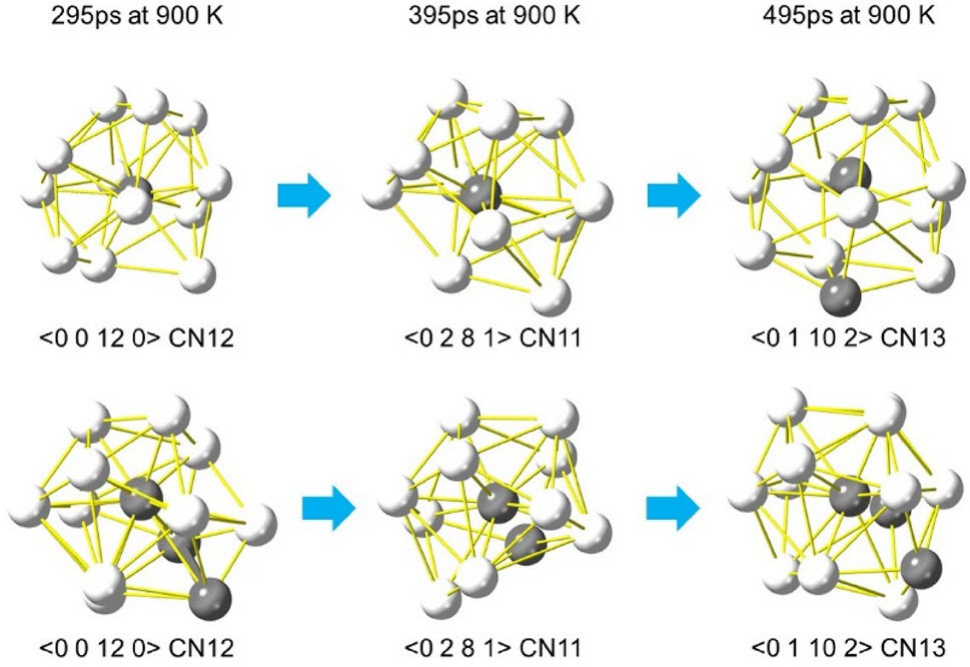
Sequential structural changes of atomic clusters during isothermal relaxation at 900 K. The relaxation process was simulated using molecular dynamics. The isothermal process was conducted after cooling from the liquid to 900 K, and the holding times at 900 K are shown above each atomic cluster. Changes in the atomic environment of atoms with the same identifiers were tracked over time. The changes in both the upper and lower atomic clusters followed the sequence <0 0 12 0>  →  <0 2 8 1>  →  <0 1 10 2> , and such transitions were indeed detected during the relaxation process.

Local PDFs for all the icosahedron-related SRO (atomic coordination polyhedra) with CN11–13, as shown in Fig. 5, exhibit broad distributions which are comparable to those of global PDFs obtained by X-ray diffraction experiment. In other words, individual SRO structures, which have a glassy disordered nature even at a sub-nanometer level, form local PDFs similar to the global PDF, regardless of coordination number. This implies that the so-called icosahedron in metallic glasses should be far from the perfect icosahedron and should be closely related to the other icosahedral-like polyhedra with different coordination numbers. In fact, the <0 1 10 2> polyhedron can be transformed into <0 0 12 0> by allowing the outermost atom to be removed as shown in Fig. 6. Similarly, <0 0 12 0> can be transformed into <0 2 8 1> . The energy barriers between the polyhedra could be discussed in the context of the local energy landscape^11^. It should be mentioned that the perfect icosahedron seen in quasicrystals^26,27^ never transforms into <0 2 8 1> or <0 1 10 2> polyhedra by removing or adding an atom (Fig. S3 in the supplementary material). The smooth linkage between the polyhedra with different coordination numbers in metallic glasses can be attributed to their better ductility than quasicrystals; moreover, this property plays a significant role in dynamic processes such as relaxation and deformation. Indeed, when we investigated the changes in atomic clusters during the structural relaxation at 900 K, which is below the glass transition temperature, we detected the sequence <0 0 12 0>  →  <0 2 8 1>  →  <0 1 10 2> , as shown in Fig. 7. This suggests that these atomic clusters easily transform into one another in the glass state. Such an imperfection of polyhedra could also be related to the origin of atomic level stresses in glasses^28^. However, the structure–property relationship in glasses based on the direct modelling remains to be solved.

The medium-range order (MRO) structures of metallic glasses are significant in relation to their mechanical properties and potential heterogeneities^29^. We are currently striving to obtain larger structures, related to possible heterogeneities using the local RMC approach. However, the current approach often leads to computational challenges, such as difficulties in eliminating overlapping atoms, highlighting the need for further development. In parallel, we are developing a complementary method utilizing virtual angstrom-beam electron diffraction (ABED) to identify MRO structures within MD simulations^30,31^. By integrating these two approaches, we aim to achieve a more comprehensive understanding of the MRO structures in metallic glasses. This combination of methodologies is expected to significantly enhance our ability to study the structures and properties of glassy materials in detail.

Finally, we briefly discuss the limitations of the local RMC modelling used in this study. It must be emphasized that it is currently impossible to quantitatively match the experimental diffraction intensities with those calculated from atomic clusters. In this work, we only discuss the positions in reciprocal space and the relative intensities of the diffraction peaks. This is because the atomic clusters are not solely responsible for generating the diffraction intensities of ABED patterns. Instead, the surrounding atoms may contribute to enhancing the diffraction intensities. Additionally, quantitative intensity measurements are not technically feasible. Furthermore, the fixed spherical boundaries used in this study may introduce biases to the results, and this aspect should be further examined. While these are challenges for future work, the significance of this study lies in deriving possible ABED patterns from a limited set of atoms.

In summary, we investigated icosahedron-related SROs in a Zr–Pt metallic glass through local RMC modelling and the local PDF analysis based on the ABED experiment. The distorted icosahedral SRO models are almost reproduced by the local RMC modelling, regardless of the initial configurations. The standard deviation of the bond distances in the SRO models obtained by local RMC are in the range 0.23–0.32 Å. Furthermore, the SRO models with coordination numbers from 11 to 13 are able to reproduce the identical ABED data. These facts imply that the SRO models satisfying the ABED data can be determined within a specific range. The features of the obtained SRO models are well consistent with those frequently found in the classical MD models. Local RMC modelling, where the structure models are directly derived from the ABED data, supports the potential-based MD simulation and vice versa, and also helps us to interpret diffraction intensity in the ABED data. Additionally, local PDF analyses can provide smooth PDF profiles for the SRO models with 11–13 coordination numbers, which are well fit to the global PDF profile. This implies that the icosahedral SRO models proposed here have a disordered nature even at the SRO level.

## Methods

### Molecular dynamics simulation

The models of amorphous Zr_80_Pt_20_ alloys were constructed using the MD method. Embedded atom method (EAM) potentials developed by Sheng were employed^32^. A cubic cell including 9600 Zr atoms and 2400 Pt was prepared with periodic boundary conditions. The initial configurations were kept at 2,500 K for 50 ps and subsequently cooled to 300 K at a cooling rate of 1.7 × 10^10^ K/s. The system temperature and pressure were controlled using a Nosé–Hoover thermostat and Nosé–Hoover pressure barostat, respectively.

### Angstrom-beam electron diffraction (ABED) experiment

A JEOL JEM-2100F transmission electron microscope with double spherical aberration correctors (operated at 200 kV) was utilised for the ABED measurements. All the ABED patterns were recorded using a CCD camera (Gatan, ES500W). A semi-parallel electron beam was produced with a specially designed small condenser lens aperture of 5 μm diameter. The convergence angle was estimated to be 3.3 mrad with a beam size of 0.4 nm. A large number of ABED patterns (more than 10,000 frames) were acquired from a thin film prepared by the ion-milling method with a cooling stage.

### Local reverse Monte Carlo modelling

A local RMC modelling optimised for ABED experiments that basically follows the steps of a conventional RMC simulation^22–24^ was developed by our group^15^. In this case, as shown in Fig. 1, a small number of atoms (12–14) were initially set inside a spherical boundary with a radius *R*. The *R* value was set to be 3.0 Å in this study. The atoms were randomly moved one by one to reduce the difference between the experimental and calculated ABED intensities. The maximum atomic displacement was set at 2.0 Å. Atomic displacements deviating beyond the boundary was rejected. The fitting ranges were − 62.8 nm^-1^ ≤ *Q*_*x*_ ≤ 62.8 nm^−1^ and − 62.8 nm^−1^ ≤ *Q*_*y*_ ≤ 62.8 nm^−1^ (41 × 41 pixels) in reciprocal space, centred on the origin. Note that *Q*_*x*_ and *Q*_*y*_ represent the magnitudes of the x and y components of the scattering vector, respectively. The judging function is the sum of the square of the diffraction intensity differences between experiment and calculation at each pixel. Some specific constraints for the atomic movements and distances were also applied. The ABED patterns were simulated via a conventional multislice method^33^.

### Local pair distribution function analysis

The pair distribution function (PDF) of each local structure was obtained by following the procedure given below. The bond lengths of all the pairs of atoms were computed for each local structure, e.g. the total bond length for CN12 was calculated to be 78. The issue of the double-count was avoided by considering a pair of atoms as a combination of two atoms. Conventionally, the PDF is obtained by considering the histogram of bond lengths to be a radial distribution function (RDF). However, we obtained the RDF based on a kernel density estimation, which is a way to estimate the probability distribution of a random variable in statistics. A Gaussian function was employed as the kernel. The bandwidth of the Gaussian function, corresponding to the bin width in the histogram, was optimised by the criterion proposed by Shimazaki and Shinomoto^34^. The 95% confidence interval of each RDF was calculated using the bootstrap method^35^. The above calculations were performed using the MATLAB code uploaded on the internet^36^. Finally, the PDF of each local structure was obtained by multiplying the RDF and the coordination number over the surface area with the radius of the bond length. The 95% confidence interval of each PDF was also obtained in the same manner.

### Supplementary Information

Supplementary Information.

## Data Availability

The data that support the findings of this study are available from the corresponding author upon reasonable request.
