# Supplementary material for: Local structural modelling and local pair distribution function analysis for Zr–Pt metallic glass
Source: Sci Rep. 2024 Jun 10;14:13322. doi: 10.1038/s41598-024-64380-2 (PMC11164872; doi:10.1038/s41598-024-64380-2)
Supplement: Supplementary file 1 — Supplementary Information. [file 41598_2024_64380_MOESM1_ESM.docx]

# Supplementary material

# Local structural modelling and local pair distribution function analysis for Zr-Pt metallic glass

Akihiko Hirata^1,2,3,4,5*^, Satoru Tokuda^6,7^, Chihiro Nakajima^8^, and Siyuan Zha^1,2^

*1. Department of Materials Science, Waseda University, Shinjuku, Tokyo, 169-8555, Japan*

*2. Kagami Memorial Research Institute for Materials Science and Technology, Waseda University, Shinjuku, Tokyo, 169-0051, Japan*

*3. WPI Advanced Institute for Materials Research, Tohoku University, Sendai, Miyagi 980-8577, Japan*

*4. Mathematics for Advanced Materials-OIL, AIST, Sendai, Miyagi 980-8577, Japan*

*5. Center for Basic Research on Materials, National Institute for Materials Science, Tsukuba, 305-0047, Japan*

*6. Research Institute for Information Technology, Kyushu University, Kasuga, Fukuoka 816-8580, Japan*

*7. Institute of Mathematics for Industry, Kyushu University, Fukuoka 816-8580, Japan.*

*8. Faculty of Science and Technology, Tohoku Bunka Gakuen University, Sendai 980-8551, Japan*

* Corresponding author

Table S1 Atomic coordinates of an icosahedral atomic cluster before and after local RMC modelling (Fig. S2). Atomic displacements for each atom are also shown.

| atom | x_before(Å) | y_before(Å) | z_before(Å) | x_after(Å) | y_after(Å) | z_after(Å) | displacement(Å) |
| --- | --- | --- | --- | --- | --- | --- | --- |
| atom1 | -0.010 | -0.012 | -3.000 | 0.416 | -0.062 | -3.126 | 0.448 |
| atom2 | -2.662 | 0.013 | -1.383 | -2.766 | -0.147 | -1.553 | 0.256 |
| atom3 | 2.203 | 1.532 | -1.343 | 1.931 | 1.790 | -1.105 | 0.444 |
| atom4 | -0.790 | 2.567 | -1.336 | -0.838 | 2.470 | -1.393 | 0.122 |
| atom5 | 2.181 | -1.574 | -1.330 | 1.827 | -1.900 | -1.593 | 0.549 |
| atom6 | -0.827 | -2.567 | -1.320 | -0.694 | -1.990 | -1.178 | 0.608 |
| center atom | 0.000 | 0.000 | 0.000 | -0.252 | -0.280 | -0.193 | 0.423 |
| atom7 | 0.827 | 2.567 | 1.320 | 0.812 | 2.484 | 1.435 | 0.142 |
| atom8 | -2.181 | 1.574 | 1.330 | -1.701 | 1.480 | 1.525 | 0.526 |
| atom9 | 0.790 | -2.567 | 1.336 | 1.137 | -1.944 | 1.066 | 0.763 |
| atom10 | -2.203 | -1.532 | 1.343 | -2.656 | -0.794 | 1.595 | 0.902 |
| atom11 | 2.662 | -0.014 | 1.384 | 2.239 | 0.092 | 1.180 | 0.481 |
| atom12 | 0.010 | 0.012 | 3.000 | -0.183 | 0.249 | 3.264 | 0.404 |


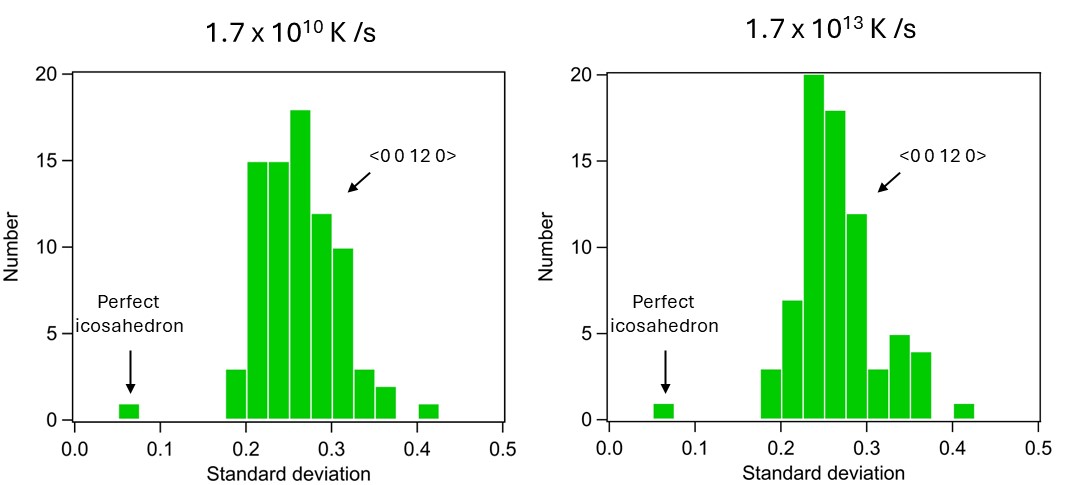


Fig. S1 Distributions of the standard deviation for interatomic distances in icosahedra extracted from Zr_80_Pt_20_ metallic glass models with varying cooling rates (left: 1.7 × 10^10^ K/s, right: 1.7 × 10^13^ K/s).


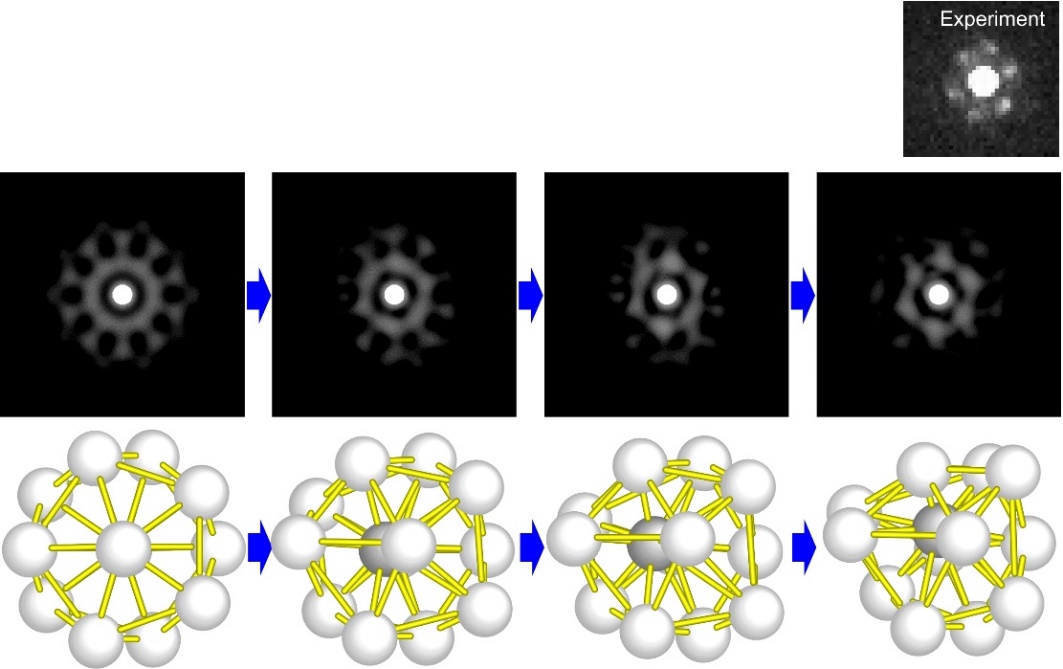


Fig. S2 Local RMC modelling started from a perfect icosahedron with small atomic displacements (less than 0.1Å)


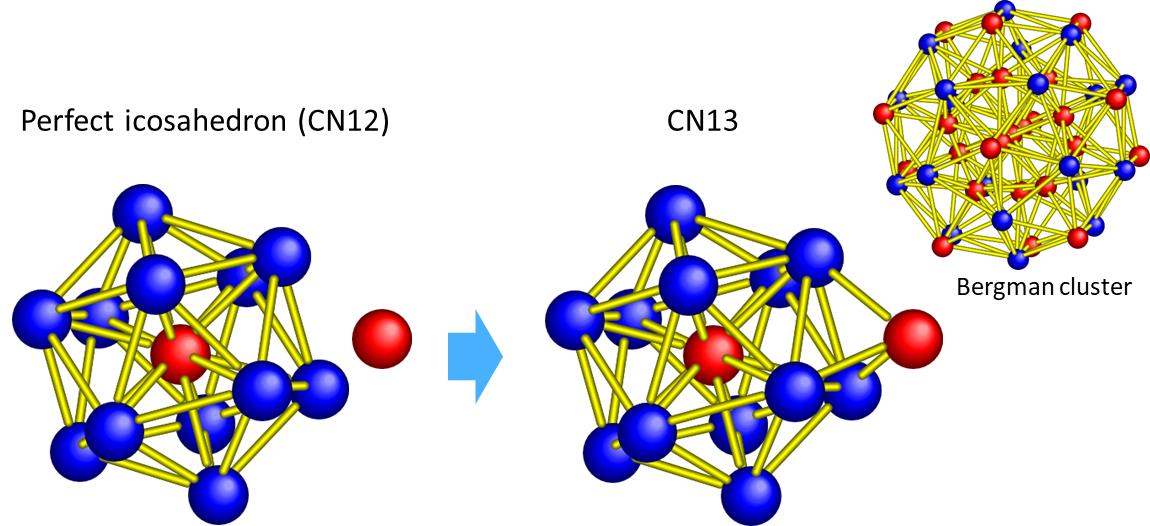


Fig. S3 Addition of an atom to the perfect icosahedron. Possible atomic sites for the added atom are just on the triangle configurations which are a part of the icosahedron. The triangle configuration makes up a tetrahedron with the added atom. The possible sites belong the second nearest neighbor positions (*see* Bergman cluster) which are definitely different from the first nearest neighbor sites.
